# Visual fields after anti-vascular endothelial growth factor therapy for retinopathy of prematurity

**DOI:** 10.1371/journal.pone.0322941

**Published:** 2025-05-07

**Authors:** Kazuki Imai, Shumpei Obata, Riko Matsumoto, Ayaka Nishida, Maki Iwasa, Masashi Kakinoki, Osamu Sawada, Yoshitsugu Saishin, Masahito Ohji

**Affiliations:** Department of Ophthalmology, Shiga University of Medical Science, Otsu, Japan; Akita University: Akita Daigaku, JAPAN

## Abstract

It is known that laser photocoagulation for retinopathy of prematurity (ROP) can cause visual field defects. There are no reports comparing the visual fields of ROP patients treated with anti-vascular endothelial growth factor (anti-VEGF) therapy and those of normal controls. A retrospective cohort study was conducted for the anti-VEGF therapy group and a prospective study was carried out for the normal control group. Visual fields were tested using Goldmann perimetry. The viewing angles in eight directions (upper, nasal-upper, nasal, nasal-lower, lower, temporal-lower, temporal and temporal-upper) and the total angle were compared between the two groups. Children aged 4 years and older who could undergo a visual field test were included. The anti-VEGF therapy group comprised children treated for Type 1 ROP with intravitreal injections of bevacizumab or ranibizumab between April 2010 and September 2019. The normal control group comprised children with best-corrected visual acuity of 1.0 or better, and without a history of any ophthalmologic diseases that cause visual field defects. Thirteen eyes of 7 patients in the anti-VEGF therapy group and 10 eyes of 5 patients in the normal control group met the criteria. The visual field angles were significantly narrower in the anti-VEGF therapy group, compared with the normal control group, for the total, and the upper, nasal-upper, nasal-lower, lower, temporal-lower, temporal and temporal-upper directions (502 versus 540 degrees, P = 0.002; 53 versus 57 degrees, P = 0.02; 55 versus 62 degrees, P = 0.04; 56 versus 61 degrees, P = 0.005; 60 versus 66 degrees, P = 0.005; 72 versus 77 degrees, P = 0.04; 82 versus 90 degrees, P = 0.005; and 62 versus 72 degrees, P = 0.002, respectively). Patients with ROP may exhibit narrower-than-normal visual fields after anti-VEGF therapy.

## Introduction

Retinopathy of prematurity (ROP) is a disease that can lead to serious visual impairment in preterm infants owing to the immaturity of the retinal vessels. Vascular endothelial growth factor (VEGF) plays a crucial role in the pathogenesis of ROP [1]. Retinal angiogenesis begins at the optic nerve at around 16 weeks gestation, with vessels extending toward the peripheral retina. Angiogenesis of the nasal retina is complete by 36 weeks, and that of the temporal retina is complete by 40 weeks. Consequently, preterm infants have underdeveloped retinal vessels, resulting in peripheral avascular areas [2]. The presence of such avascular areas causes hypoxia in the retina after birth, leading to the growth of neovascular vessels into the vitreous, due to an overproduction of VEGF. If left untreated, this condition can progress to retinal detachment and vision loss [3].

Laser photocoagulation has traditionally been considered to be the gold standard treatment for ROP. It destroys the peripheral avascular retina, inducing VEGF production, and may suppress disease progression; however, it can cause visual field defects [2,4]. McLoone et al. reported that patients in their laser-treatment group had narrower visual fields than did patients in the spontaneous-resolution-ROP group [4]. In contrast, anti-VEGF therapy, a recently introduced treatment modality, is expected to promote retinal revascularization after treatment [5–8] and may preserve a wider visual field as retinal vessels grow toward the peripheral retina. However, Tahija et al. reported that persistent avascular retina (PAR) could remain in the periphery without complete retinal vascularization after anti-VEGF therapy [7]. Furthermore, capillary beds in the peripheral retina might not develop sufficiently [6,9]. Retinal function in areas of revascularization after birth or after treatment has not been fully investigated.

We previously reported that an anti-VEGF therapy group had wider visual fields than a laser therapy group [10]. However, to our knowledge, there are no reports comparing the visual fields of ROP patients treated with anti-VEGF therapy and those of children acting as normal controls. The purpose of this study was to compare the visual fields between patients with ROP treated with anti-VEGF therapy and pediatric controls.

## Materials and methods

### Study design

This study received approval from the Institutional Review Board and Ethics Committee of Shiga University of Medical Science Hospital and was conducted in accordance with the ethical standards of the hospital, the national research committee, and the Helsinki Declaration. An opt-out approach was implemented for the retrospective study in the anti-VEGF therapy group, whereas consent was obtained for the prospective study in the control group.

### Patients

Children aged 4 years and older who could undergo a visual field test at Shiga University of Medical Science Hospital were included. The anti-VEGF therapy group comprised children treated for Type 1 ROP with intravitreal injections of bevacizumab (IVB) or ranibizumab (IVR) between April 2010 and September 2019. Eyes with a history of laser photocoagulation in addition to the anti-VEGF therapy were excluded. Type 1 ROP was defined according to the ETROP study [11].

For IVB treatment, consent for off-label use was obtained from the parents and approval was secured from the Ethics Committee of Shiga University of Medical Science Hospital. For IVR treatment, consent for the clinical trial for approval of ranibizumab for ROP by Japanese Government was obtained. The anti-VEGF therapy was performed after obtaining informed consent from the parents, following explanation of the risks and benefits of anti-VEGF therapy as well as the laser treatment.

IVB (0.625 mg/ 0.025 mL) was administered with a 29-gauge needle and IVR (0.2 mg/ 0.02 mL) was administered with a 30-gauge needle through the pars plana, 0.75- to 1.0-mm posterior to the corneal limbus.

We accessed the data for research purposes in 10/1/2023–1/2/2023, 27/7/2024 and 19/12/2024. We did not have access to information that could identify individual participants during or after data collection. We have created anonymized information by giving research subjects research IDs that do not identify individuals on their own. The information obtained in the research is anonymized by managing it using the research ID.

The control group comprised children with best-corrected visual acuity of 1.0 or better, and without a history of any ophthalmologic diseases that cause visual field defects. The allowed range of refractive error was spherical refraction from −2.5 diopters to + 1.5 diopters, with astigmatism less than 1.5 diopters. Refractive error was defined according to the Pediatric Eye Evaluations Preferred Practice Pattern by the American Academy of Ophthalmology [12]. The examination of children in the control group was performed after obtaining informed consent document from the parents from 27/1/2023–11/4/2023.

### Examination

A visual acuity test, a measurement of refraction and a visual field test using Goldmann perimetry were conducted. The Goldmann perimetry was performed with a background luminance of 31.5 apostilb, a target luminance of 1,000 apostilb and a target size of V4e (64 mm^2^). The visual field angle was compared between the anti-VEGF therapy group and the control group, along eight directions: upper, nasal-upper, nasal, nasal-lower, lower, temporal-lower, temporal and temporal-upper. The total viewing angle, summed for the eight directions, was also compared between the two groups.

### Statistical analysis

Statistical analysis was performed with “Easy R” (EZR) [13]. The Mann-Whitney U test was used to compare continuous variables. *P*-values less than 0.05 were considered statistically significant.

## Results

### Baseline characteristics

During the study period, thirteen eyes of 7 patients in the anti-VEGF therapy group and 10 eyes of 5 patients in the control group met the inclusion criteria. No significant differences were observed between groups in the male/female ratio (9/4 versus 4/6, *P* = 0.18) or age at examination (median: 4.9 (4.1–6.8) versus 5.2 (4.2–6.2) years, *P* = 0.51). However, logarithm of the minimum angle of resolution (logMAR) visual acuity was worse in the anti-VEGF group than in the control group (median: 0.15 versus −0.079 logMAR, *P* = 0.008) and the equivalent spherical refraction was more myopic in the anti-VEGF group than in the control group (median: −1.0 versus 0.13 diopters, *P* = 0.04). All data are provided in S1 File.

The zone and stage of ROP, the presence of plus disease, the treatment details and the baseline characteristics in the anti-VEGF therapy group are presented in Table 1. Six eyes were in Zone I (including two eyes of AP-ROP (aggressive posterior retinopathy of prematurity)); seven eyes were in Zone II; and no eyes were in Zone III at the time of treatment. IVB was performed in nine eyes and IVR was performed in the other four eyes. The interval between IVR/IVB treatment and visual field testing was a median of 4.8 (3.8–6.6) years. Both eyes of one patient underwent two IVR cycles. All complications are being managed and treated appropriately under the care of pediatric specialists.

**Table 1:** Conditions and treatments of the eyes, and baseline characteristics in the anti-VEGF therapy group.

Case	Zone ICROP2		Stage		Plus	PMA at Tx, week			Tx	
1-R	2		3		pre plus	35.3			IVB	
1-L	2		3		pre plus	35.3			IVB	
2-R	2		3		pre plus	36.0			IVB	
3-R	2		3		pre plus	34.3			IVB	
3-L	2		3		pre plus	34.3			IVB	
4-R	1		3		pre plus	37.7			IVR	
4-L	1		3		pre plus	37.7			IVR	
5-R	1		3		plus	33.6			IVR, twice	
5-L	1		3		plus	33.6			IVR, twice	
6-R	2		3		pre plus	39.0			IVB	
6-L	2		3		pre plus	39.0			IVB	
7-R	AP-ROP					34.0			IVB	
7-L	AP-ROP					34.0			IVB	
Case	GA, week	BW, g	AS1	AS5	RDS	BPD	PDA	IVH	NEC	Sepsis
1	25.7	778	5	10	Yes	Yes	No	Yes	No	No
2	26.1	1138	8	4	Yes	Yes	Yes	Yes	No	No
3	26.1	704	7	9	Yes	Yes	Yes	Yes	No	No
4	26.3	710	6	9	Yes	Yes	Yes	No	Yes	Yes
5	24.0	654	4	9	Yes	Yes	Yes	Yes	No	Yes
6	24.4	449	4	9	Yes	Yes	Yes	No	No	No
7	28.0	1030	1	4	Yes	Yes	Yes	No	No	No
Case	NA	NJ	Other systemic complications							
1	Yes	Yes	LCC, VSD							
2	Yes	Yes	PPS							
3	Yes	No	HT, LCC, inguinal hernia, kawasaki disease, testicular atrophy							
4	Yes	Yes	FIP, GHD, HT, LCC, SVCS, bronchial asthma, febrile seizure							
5	Yes	No	cryptorchidism							
6	Yes	No	DLS, PPHN, hip abduction restriction, meconium ileus							
7	Yes	No	CAF, DLS, LCC, PPHN, pneumothorax							

VEGF: vascular endothelial growth factor, ICROP: International Classification of Retinopathy of Prematurity, PMA: postmenstrual age, Tx: treatment, IVB: intravitreal injection of bevacizumab, IVR: intravitreal injection of ranibizumab, R: right eye, L: left eye, AP-ROP: aggressive posterior retinopathy of prematurity, GA: gestational age, BW: birth weight, AS1: Apgar score at one minute, AS5: Apgar score at five minutes, RDS: respiratory distress syndrome, BPD: bronchopulmonary dysplasia, PDA: patent ductus arteriosus, IVH: intraventricular hemorrhage, NEC: necrotizing enterocolitis, NA: neonatal anemia, NJ: neonatal jaundice, LCC: late-onset circulatory collapse, VSD: ventricular septal defect, PPS: peripheral pulmonary stenosis, HT: hypothyroidism, FIP: focal intestinal perforation, GHD: growth hormone deficiency, SVCS: superior vena cava syndrome, DLS: dry lung syndrome, PPHN: persistent pulmonary hypertension of the newborn, CAF: coronary artery fistula.

### Visual field tests

The composite results of the visual field tests are presented in Table 2. The visual field angles for the total of eight directions were smaller in the anti-VEGF group than in the control group (median: 502 versus 540 degrees, *P* = 0.002). The visual field angles were smaller for all directions in the anti-VEGF group than in the control, with statistically significant differences in the upper, nasal-upper, nasal-lower, lower, temporal-lower, temporal and temporal-upper directions (median: 502 versus 540 degrees, *P* = 0.002; 53 versus 57 degrees, *P* = 0.02; 55 versus 62 degrees, *P* = 0.04; 56 versus 61 degrees, *P* = 0.005; 60 versus 66 degrees, *P* = 0.005; 72 versus 77 degrees, *P* = 0.04; 82 versus 90 degrees, *P* = 0.005; and 62 versus 72 degrees, *P* = 0.002, respectively).

**Table 2:** Visual field sizes; medians and ranges.

Direction	Anti-VEGF therapy Visual field sizes, degree Median (range)	Control Visual field sizes, degree Median (range)	*P* value
Upper	53 (46-58)	57 (49-61)	0.02
Nasal-upper	55 (50-63)	62 (48-64)	0.04
Nasal	59 (54-62)	60 (52-64)	0.30
Nasal-lower	56 (51-62)	61 (57-70)	0.005
Lower	60 (53-72)	66 (60-71)	0.005
Temporal-lower	72 (53-80)	77 (70-88)	0.04
Temporal	82 (71-90)	90 (81-91)	0.005
Temporal-upper	62 (54-75)	72 (65-85)	0.002
Total	502 (445-537)	540 (499-570)	0.002

VEGF: vascular endothelial growth factor.

The visual field measurements for each eye, along each test direction, are presented in Table 3.

**Table 3:** Visual field measures (degrees) for all eyes in the study, by direction.

Case	Upper	Nasal-upper	Nasal	Nasal-lower	Lower	Temporal-lower	Temporal	Temporal-upper	Total
Anti-VEGF therapy									
1-R	52	53	58	53	60	72	71	59	478
1-L	55	51	55	52	59	65	77	60	474
2-R	52	55	60	59	62	71	84	65	508
3-R	48	55	55	51	56	68	80	57	470
3-L	48	56	59	55	63	73	82	55	491
4-R	57	63	60	55	56	71	85	67	514
4-L	58	63	62	58	59	73	80	67	520
5-R	52	57	57	52	60	80	82	62	502
5-L	54	50	59	58	60	80	88	67	516
6-R	46	52	57	58	53	53	72	54	445
6-L	55	55	54	60	60	64	77	62	487
7-R	55	59	60	62	72	78	87	64	537
7-L	53	54	59	56	65	78	90	75	530
Control									
8-R	56	58	55	57	66	84	86	66	528
8-L	49	57	59	59	60	74	89	67	514
9-R	50	48	52	57	67	76	84	65	499
9-L	61	63	60	61	62	70	81	75	533
10-R	58	62	64	70	71	80	91	74	570
10-L	58	64	58	60	65	88	90	85	568
11-R	59	62	62	68	64	73	90	77	555
11-L	57	62	64	67	65	83	91	74	563
12-R	56	59	60	66	67	73	91	70	542
12-L	57	61	59	59	68	77	87	69	537

VEGF: vascular endothelial growth factor, R: right eye, L: left eye.

## Discussion

The current study suggests that the visual fields of ROP patients after anti-VEGF therapy may be narrower than those of normal controls at the same age, with significant differences noted at seven of the eight directions tested, as well as the sum across directions. Anti-VEGF therapy is expected to promote retinal vascularization [5–8] and may widen the visual field as retinal vessels grow toward the peripheral retina, however, these earlier reports did not examine the patients’ visual fields. Our previous report indicated that the anti-VEGF group had wider visual fields than did the laser therapy group [10]. However, to the best of our knowledge, there have not been any reports comparing visual fields between an anti-VEGF therapy ROP group and a normal control group. This study represents the first such report.

Several factors might contribute to the insufficient visual field development observed in the anti-VEGF therapy group in this study. First, a significant factor is the presence of persistent avascular retina (PAR). Following anti-VEGF therapy, vascular elongation from Zone I or Zone II to Zone III has been reported in many cases [6–8]. However, the retinal vessels of premature infants often do not achieve complete retinal vascularization up to the ora serrata even after anti-VEGF therapy, leaving PAR in the periphery [7]. Toy et al. reported that 91% of the ROP patients after IVB treatment had not reached full retinal vascular maturity by 54 weeks of gestation, and Tahija et al. reported that 55% of patients had not done so at 87.5 weeks after IVB treatment [7,14]. Ling et al. advised that anti-VEGF therapy is a risk factor for PAR in 6- to 8-year-olds and that 32% of their patients with ROP have PAR after anti-VEGF therapy [15]. Some degree of vascular elongation can be expected with anti-VEGF therapy but the vessels may not reach the ora serrata, so, PAR could remain in the patients after anti-VEGF therapy for ROP. Although the details of PAR are not certain in the present study, owing to the absence of fluorescein angiography (FA), the presence of PAR in the anti-VEGF group could have contributed to peripheral retinal dysfunction and narrowing of the visual fields. The existence of PAR also remains a clinical concern, owing to the risk of late complications, such as retinal detachment [16].

Another contributing factor could be underdevelopment of the capillary beds in the peripheral retina. Lorenz et al. [6] and Lepore et al. [17] used FA to assess retinal vascular development after anti-VEGF therapy. Both groups reported that the capillary beds may not fully develop in the peripheral retina despite retinal vascular growth, so the retina may not function properly. Lorenz et al. found a loss of capillary beds in follow-up examinations up to 45 months after treatment [6]. Likewise, Lepore et al. observed that 75% of patients still lacked capillary beds even 4 years after anti-VEGF therapy [17]. Mansukhani et al.’s comparison between an anti-VEGF therapy group, and a group of patients with ROP that had spontaneously regressed, revealed that both groups exhibited abnormal capillary beds in the peripheral retina [18]. Although there are some controversial reports about the development of the capillary beds in the peripheral retina, abnormal capillary beds could influence the development of visual fields.

The visual fields in patients with ROP were narrower than those of normal controls, except for the nasal visual field. The nasal visual field corresponds to the temporal retina, where retinal vessel formation is completed last. In patients with ROP, the nasal visual field reached the normal values observed in adults, suggesting that it had reached a ceiling, thereby resulting in no significant difference. One possible explanation for this finding is that the nasal visual field is inherently narrower than the temporal visual field due to the anatomical presence of the nose. Consequently, it may have been less affected by temporal retinal vessel formation. On the other hand, a larger sample size may increase the possibility of achieving statistical significance.

This study has several limitations. First, the sample size was relatively small. Second, we did not perform FA in this study, so the detailed retinal vascular development in these cases is unknown. Third, the long-term course of vision in these patients has not been investigated.

In conclusion, our findings suggest that patients with ROP who undergo anti-VEGF therapy may exhibit narrower visual fields compared with normal controls. Further research, including long-term follow-up, is warranted as anti-VEGF therapy has emerged as the primary treatment for ROP.

## Supporting information

S1 File Data set for analysis. (XLSX)
